# Poldip2 deficiency attenuates lung disease severity in a mouse model of COVID-19

**DOI:** 10.1371/journal.pone.0348065

**Published:** 2026-04-29

**Authors:** Ruinan Hu, Alejandra Valdivia, Taylor White, Willy Ju, Maegan L. Brockman, Zhan Zhang, Hongyan Qu, Georgette Gafford, Giji Joseph, Samantha Burton, Leda Bassit, Tysheena P. Charles, Raymond F. Schinazi, Rebecca D. Levit, Cynthia A. Derdeyn, Kathy K. Griendling, Bernard Lassègue, Marina S. Hernandes

**Affiliations:** 1 Emory University School of Medicine, Department of Medicine, Division of Cardiology, Atlanta, Georgia, United States of America; 2 Emory University School of Medicine, Department of Pathology and Laboratory Medicine, Atlanta, Georgia, United States of America; 3 Emory National Primate Research Center, Emory University, Atlanta, Georgia, United States of America; 4 Center for ViroScience and Cure, Laboratory of Biochemical Pharmacology, Department of Pediatrics, Emory University School of Medicine and Children’s Healthcare of Atlanta, Atlanta, Georgia, United States of America; 5 University of Washington, School of Medicine, Department of Laboratory Medicine and Pathology, Seattle, Washington, United States of America; University of Coimbra: Universidade de Coimbra, PORTUGAL

## Abstract

The lungs are the primary target of severe acute respiratory syndrome coronavirus 2 (SARS-CoV-2), with the infection resulting in lung inflammation, pulmonary vascular leakage and diffuse alveolar damage. Polymerase delta-interacting protein-2 (Poldip2) mediates lung inflammation and vascular permeability after lipopolysaccharide-induced acute respiratory distress syndrome; however, whether it also affects the pathological consequences of SARS-CoV-2 infection is completely unknown. Here, we assessed the role of Poldip2 in inflammation, immune cell infiltration and lung tissue damage in response to SARS-CoV-2. Our data show that Poldip2 expression was elevated in human lung vascular endothelium after infection. In a Poldip2-deficient heterozygous mouse model, acute clinical symptoms were not affected. However, seven days after infection, Poldip2 knockdown reduced viral load, decreased infiltration of myeloperoxidase (MPO)-positive neutrophils into inflamed lungs, and reduced tissue damage. Poldip2 also modulated the inflammatory response to viral infection in a heterogeneous manner, reflecting its diverse regulatory roles. These data support the concept that targeting Poldip2 could potentially attenuate severe lung injury following SARS-CoV-2 infection.

## Introduction

Severe acute respiratory syndrome coronavirus-2 (SARS-CoV-2) initiated the global Coronavirus Disease 2019 (COVID-19) pandemic. According to the World Health Organization, as of August 2025, more than 760 million COVID-19 cases and 7 million deaths have been reported worldwide [1]. SARS-CoV-2 is primarily transmitted through respiratory droplets and infects human cells via binding to angiotensin-converting enzyme 2 (ACE2), which is the main SARS-CoV-2 receptor [2]. Early in infection, SARS-CoV-2 has been shown to target nasal and bronchial epithelial cells and pneumocytes, resulting in high copy numbers in the lower respiratory tract [3]. Pro-inflammatory signaling molecules are released by infected cells and alveolar macrophages, which then trigger the recruitment of T lymphocytes, monocytes, and neutrophils, contributing to further aggravating the inflammatory response. In the late stages of infection, damage to vascular endothelial cells and alveolar epithelial cells compromises the epithelial-endothelial barrier integrity, leading to vascular leakage and pulmonary edema [3], with accumulation of fluid containing large amounts of proteins and cellular debris, hyaline membrane formation [4–7] and early-phase acute respiratory distress syndrome [3].

Additional pathophysiological mechanisms associated with SARS-CoV-2 infection include an acute inflammatory response which was found to be positively correlated with the severity of COVID-19 symptoms [8,9]. Evaluation of bronchoalveolar lavage (BAL) fluid collected from COVID-19 patients revealed high levels of pro-inflammatory cytokines including IL-1β, IL-8 and TNFα [10], suggesting that lung inflammation is a major component of SARS-CoV-2 pathogenesis. Corroborating these findings from human samples, SARS-CoV-2 infection has been shown to induce TNFα, IFN-γ, MCP1 and IL-6 protein expression in the lung tissue of humanized ACE2 transgenic mice [11]. In a different study, a liquid chromatography-mass spectrometry-based proteomic and phosphoproteomic evaluation of lung tissue samples isolated from SARS-CoV-2 infected K18-hACE2 mice showed the presence of hyperphosphorylated proteins associated with leukocyte transendothelial migration [12]. The same study also demonstrated enhanced phosphorylation of tight junction proteins in lung tissue in response to SARS-CoV-2 infection [12], which is a critical mechanism involved in endothelial barrier dysfunction [13] and acute lung injury [4].

Polymerase δ-interacting protein 2 (Poldip2) was originally identified as a binding partner of polymerase-δ and was proposed to be involved in DNA damage repair [14,15]. In more recent studies, our lab discovered that Poldip2 is a critical regulator of inflammation and endothelial barrier function [13,16–18]. Using a lipopolysaccharide (LPS)-induced acute respiratory distress syndrome (ARDS) model, our group has recently reported that Poldip2 knockdown decreases cytokine and chemokine induction and lung immune cell infiltration [18,19]. Our *in vitro* studies further demonstrated that Poldip2 downregulation in pulmonary microvascular endothelial cells attenuates the protein expression of leukocyte adhesion molecules and decreases LPS-induced THP-1 monocyte adherence [19], suggesting that Poldip2 plays a role in lung endothelial cells during inflammation. In subsequent studies we demonstrated that Poldip2 depletion in pulmonary microvascular endothelial cells prevents disruption of VE-cadherin, one of the main endothelial cell junction proteins, and the subsequent increase in permeability in response to TNFα, likely in a Rho pathway-dependent manner [18]. Taken together, these results suggested an important role for Poldip2 in inflammation and endothelial barrier function during ARDS.

Based on these observations, we hypothesized that depletion of Poldip2 would also be protective against SARS-CoV-2-induced lung injury. To test this hypothesis, we crossed K18-humanized ACE2 transgenic mice with Poldip2^+/-^ mice. Following intranasal inoculation with SARS-CoV-2, disease severity, lung tissue damage, inflammation and immune cell infiltration were evaluated. While heterozygous deletion of Poldip2 does not affect the susceptibility to SARS-CoV-2 infection, our data indicate that Poldip2^+/-^ mice exhibit decreased infiltration of myeloperoxidase (MPO)-positive neutrophils, reduced lung tissue damage and a complex modulation of inflammatory mediators. These data suggest that targeting Poldip2 may be beneficial in case of SARS-CoV-2 infection.

## Materials and methods

### Animals

K18-hACE2 transgenic (strain # 034860) and C57BL/6J (strain # 000664) mice were purchased from The Jackson Laboratory. Genetic drift was minimized by backcrossing K18-hACE2 to C57BL/6. Genotyping was performed either using a traditional 4 primer PCR assay (**Table 1**), or by real-time PCR when it was necessary to detect homozygotes. In the latter case, the assay took advantage of an SNP adjacent to the ACE2 transgene. It was performed with a QuantStudio 7 instrument (Thermo Fisher), Luna Universal Probe qPCR Master Mix (M3004L, New England Biolabs) and two primers spanning 220 bp. Wild type and mutant alleles were respectively detected using FAM and HEX labeled TaqMan probes (Millipore Sigma) (**Table 1**). Annealing and extension were carried out at 58°C for 40 seconds. Constitutive Poldip2 knockout mice were produced and genotyped as described previously [20] or using a real-time PCR assay based on high-resolution melting curve analysis (**Table 1**). Experimental animals were generated by crossing homozygous ACE2 transgenics with Poldip2^+/-^ mice to obtain equal numbers of heterozygous ACE2 transgenics with either Poldip2^+/-^ or Poldip2^+/+^ (littermate controls) genotypes. In all experiments, male and female human ACE2 and Poldip2 heterozygous mice and littermate controls, aged 3–5 months were randomly assigned to control and experimental groups using a random number generator. The number of female and male animals in each group was approximately similar. All methods involving live animals were carried out in compliance with the ARRIVE guidelines [21]. Mice were euthanized with a standard CO_2_ inhalation protocol following the latest recommendations of the American Veterinary Medical Association. All animal welfare and experimental protocols were approved by the Emory University Institutional Animal Care and Use Committee. Ethical approval was received before conducting the study. Researchers performing analyses were blinded to genotype and experimental groupings of mice.

**Table 1:** Primer and probe sequences.

Target	Type	Sequence 5’ to 3’
**Genotyping assays**		
Human ACE2 Tg Mouse GAPDH (internal control) (regular assay)	Forward 1 Reverse 1 Forward 2 Reverse 2	ACCTGGCTGAAAGACCAGAACAAG TCAAATTAGCCACTCGCACA CTCCCAACCCCAGAGGTAGT AGACCCCAGATCCAGAAAGG
Human ACE2 Tg [+N] = locked nucleic acid (real-time assay)	Forward Reverse Probe 1 Probe 2	CATCCGGGTTTTAATAATGCT TTCGCACACTTCTGTCAA 6-FAM/AAC[+T]GC[+T]TT[+T]CA[+A]ATGCTA/BHQ1 HEX/AAC[+T]GC[+T]TT[+G]CA[+A]ATGCTA/BHQ1
Mouse Poldip2+/- (regular assay)	Forward Reverse 1 Reverse 2	CTTCTTGGTTTCTTGATGCACAGT GTTTTGCCTTTCACCTCCTTAGAGCC ACATTATACGAAGTTATGTACGCCTA
Mouse Poldip2+/- (real-time assay)	Forward 1 Reverse 1 Forward 2 Reverse 2	GCAGAAGCCCCTTCAGATCA GTTTTGCCTTTCACCTCCTTAGAGC CTGGAGCTAGCAGACAAAGTCCCT GCCGACTAGGCCATCTTTGATATCTCG
**RT-qPCR assays**		
SARS-CoV-2 N	Forward Reverse Probe	ATGCTGCAATCGTGCTACAA GACTGCCGCCTCTGCTC 6-FAM/TCAAGGAAC/ZEN/AACATTGCCAA/IABkFQ
SARS-CoV-2 sgE	Forward Reverse Probe	ACCTTCCCAGGTAACAAACCA AACTAGCAAGAATACCACGAAAGCA 6-FAM/TCTTGTAGA/ZEN/TCTGTTCTCTAAACGA/IABkFQ
Mouse TNF𝛼	Forward Reverse	CTATGTCTCAGCCTCTTCTC GGCCATTTGGGAACTTCTCA
Mouse IL-1β	Forward Reverse	ACCAAGCAACGACAAAATAC CACTTTGCTCTTGACTTCTATC
Mouse IL-6	Forward Reverse	CTACCCCAATTTCCAATGCT ACCACAGTGAGGAATGTCCA
Mouse MCP1	Forward Reverse	CAAGATGATCCCAATGAGTAG CAGATTTACGGGTCAACTTC
Mouse CXCL1	Forward Reverse	AAAGATGCTAAAAGGTGTCC GTATAGTGTTGTCAGAAGCC
Mouse IFN-γ	Forward Reverse	TGAAAGACAATCAGGCCATCAGCAA ACTTGGCAATACTCATGAATGCATC
Mouse Poldip2	Forward Reverse	GAGACCACCGAGAACATCCG GTGGGAATTCTGGGCTTCCCTCA
Human ACE2	Forward Reverse	ACCTGGCTGAAAGACCAGAACAAG AATTAGCCACTCGCACATCC

### Virus preparation

The SARS-CoV-2 strain (hCoV-19/US-WA1/2020) was obtained from Biodefense and Emerging Infections Research Resources Repository (Cat NR53899, Lot 7004383, BEI Resources). The virus was propagated in Vero E6/TMPRSS2 cells (passage 5, from Japanese Collection of Research Bioresources Cell Bank, Sekisui XenoTech, LLC) under 37°C/5% CO_2_, the multiplicity of infection (MOI) was 0.025. The virus was harvested after 48 hours, or when 80% cytopathic effect was observed. Virus titer was determined by 50% tissue culture infective dose (TCID_50_/ml) or plaque assays (plaque for PFU/ml). The concentration of the virus was 1.78x10^9^ TCID_50_ (calculated using the Spearman Karber method) and 1.25x10^9^ PFU/ml. Small aliquots of virus were made and stored at −80°C or in liquid nitrogen until use. All experiments involving infectious virus were conducted at Emory University in approved biosafety level 3 (BSL3) laboratories with routine medical monitoring of laboratory personnel.

### Animal model of SARS-CoV-2 infection

Female and male C57BL/6J mice with the K18-hACE2 transgene and either Poldip2^+/+^ or Poldip2^+/-^ genotypes were anesthetized with 3% isoflurane (07-894-9580, Patterson Veterinary) and intranasally infected with 4x10^5^ plaque-forming units (PFU) of SARS-CoV-2 virus diluted in 20 µl phosphate-buffered saline (PBS) in an Animal Biosafety Level 3 (ABSL3) facility at the Emory National Primate Research Center. Uninfected control mice received an equal volume of PBS. Mice were maintained in Sealsafe HEPA-filtered air in/out units for the duration of the experiments. Following infection, mice were monitored daily for visual appearance and behavior by specially trained researchers, veterinarians and veterinary technicians. Clinical symptoms were recorded and scored according to a 1–5 scale (from no symptom to moribund). DietGel Boost (72-04-5022, ClearH_2_O) was placed on the floor of the cage to facilitate access to food and hydration and small hand warmers (HotHands, Kobayashi Consumer Products) were placed under one side of the cage to minimize hypothermia. Mice were immediately humanely euthanized using standard IACUC approved procedures if certain indicators of distress were present, including a 25% weight loss, a 3°C hypothermia or a clinical score of 5. Thus, data presented in the following sections were acquired from 64 mice, while approximately 6 additional mice had to be excluded because they died or were euthanized after reaching the indicated humane endpoints. Body temperature was measured using a non-contact infrared thermometer (AdTemp 429, American Diagnostic Corporation) under light anesthesia (1–1.5% isoflurane). Body temperature and weight were recorded at baseline and every other day. Relative body temperature was calculated by dividing the temperature of each mouse by its temperature on day zero. This allowed each individual mouse to serve at its own control and reduced overall variability between animals. A similar normalization was performed with body weights, followed by multiplication by 100 to express results as a percentage. Mice were euthanized at day 7 postinfection and bronchoalveolar lavage (BAL) fluid and lung tissue were collected for the determination of inflammatory gene and protein expression, leukocyte infiltration and histological analysis. A schedule of procedures is presented in **Table 2** and a summary of group assignments in **Table 3**.

**Table 2:** Experimental timeline.

Days	Action
< 0	Moving mouse cages to the ABSL3 facility a few days before the start of the experiment.
0	Recording baseline body weights and temperatures. Nasal instillation of PBS or virus.
1-6	Monitoring mice daily for clinical signs. Recording body weights and temperatures every other day.
7	Euthanasia by CO_2_ inhalation.
7	BAL collection for cytokine protein assays. Centrifugation and inactivation of virus in supernatants at 56°C for 30 min.
7	Lung tissue collection for histology. Tracheal injection with 10% formalin. Further incubation in formalin for one week for tissue fixation and virus inactivation.
7	Lung tissue collection for RNA. Homogenization in QIAzol with TissueLyser for RNase inhibition and virus inactivation.

**Table 3:** Numbers of male (M) and female (F) mice in experimental groups.

	Poldip2 + /+				Poldip2 + /-			
	PBS		CoV		PBS		CoV	
	M	F	M	F	M	F	M	F
Fig 2	8	8	11	10	6	2	6	6
Fig 3A			3	4			4	2
Fig 3B			3	4			3	2
Fig 4	2	3	2	3	3	2	4	2
Fig 5	2	2	2	3	3	2	4	2
Fig 6A MCP1 mRNA	6	7	9	8	5	0	6	4
Fig 6A CXCL1 mRNA	5	7	7	8	4	0	6	4
Fig 6B BAL proteins	3	2	4	5	5	0	5	4
Fig 7A TNFα, IL-1β, IL-6 mRNAs	6	7	9	9	5	0	6	4
Fig 7A IFN-γ mRNA	3	3	5	6	5	0	6	4
Fig 7B IL-1β, IL-6, IFN-γ proteins	7	8	10	9	5	2	5	4
Fig 7B TNFα protein	5	6	6	5	5	0	3	2
S2 Fig	7	2			6	3		

### Determination of *in vivo* viral load in lung tissue

Lung viral load was determined at day 7 postinfection. Briefly, the lower lobes of the mouse lung were homogenized in 1 mL of QIAzol reagent (79306, Qiagen) using one stainless steel bead (3/16“Inch 440 Stainless Steel Ball Bearings G100, BC Precision), in a TissueLyser LT (85600, Qiagen) at 25 Hz for 10 min. Total RNA was purified with the RNeasy Plus kit (74134, Qiagen). Expression of the SARS-CoV-2 N gene and subgenomic E RNA were measured by RT-qPCR using primers and TaqMan probes described by Hassan et al. [22], Koh et al. [23], Chen et al. [24] (**Table 1**) and purchased from IDT. The assay was carried out with a QuantStudio 7 instrument (Thermo Fisher), using Luna Universal Probe qPCR Master Mix (M3004L, New England Biolabs) with annealing and extension at 60°C. Data quantification was performed using the qpcR software library in the R environment, followed by normalization and scaling as described in the RT-qPCR section.

### Bronchoalveolar lavage (BAL) fluid collection

After euthanasia, mouse tracheas were exposed through a small skin incision on the anterior neck and cannulated using a 21-gauge lavage needle. PBS (1.2 ml) was injected through the needle and retrieved. Following centrifugation (300 g, 10 min), the supernatant was incubated in a pre-warmed dry heat block at 56°C for 30 min to inactivate the virus and subsequently stored at −80°C.

### Chemokine and cytokine protein assays in BAL fluid

Specific mouse sandwich enzyme-linked immunosorbent assays (ELISA) were carried out using kits from R&D Systems: TNFα (MHSTA50), IL-1B/IL-1F2 (MHSLB00), IL-6 (M6000B-1), MCP1/CCL2 (MJE00B), CXCL1/KC (MKC00B-1), IFN-γ (MIF00–1). Assays were performed according to the manufacturer’s instructions. Briefly, duplicate standards or samples were added to individual microplate wells coated with a monoclonal antibody against the desired protein. Following incubation and washing, a polyclonal antibody against the same protein, conjugated to horseradish peroxidase directly or via biotin-streptavidin, was added. Enzymatic activity was measured with a microplate reader, after addition of a chromogenic substrate. Sample concentrations were calculated from a standard curve. Results were finally normalized as described in the RT-qPCR section.

### Histology and immunofluorescence

Human lung samples from the Georgia Medical Examiner’s Office were fixed in 10% formalin in neutral buffered saline (HT501128, Millipore Sigma) for one week, dehydrated using a HistoCore PEARL automatic tissue processor (Leica), transferred to 70% ethanol, embedded in paraffin and sectioned at 5 µm thickness. Following deparaffinization and antigen retrieval using citrate buffer (pH 6.0) (40125008–2, BioWorld) in a pressure cooker for 10 min, sections were blocked with 2% IgG-free BSA (001-000-162, Jackson ImmunoResearch) and 5% normal goat serum (5425S, Cell Signaling) in PBS for 1 hour at room temperature. Primary antibodies against Poldip2 (rabbit, HPA007700, Sigma Aldrich) and CD31 (mouse, Ab9498, Abcam), or with the corresponding IgG isotype controls (ab18450 and ab172730, Abcam) were incubated overnight at 4°C. After washing with PBS, sections were incubated with anti-rabbit Alexa FluorTM 568 (A-11011, Invitrogen) and anti-mouse Alexa FluorTM 488 (A-11001, Invitrogen) secondary antibodies for 1 hour at room temperature. Nuclei were counterstained with DAPI (D9542, Sigma-Aldrich), and slides were mounted using Diamond Antifade (P36961, Thermo Fisher Scientific).

Images were acquired as z-stack with a Zeiss LSM800 Airyscan laser scanning confocal microscope with a Plan-Apo 63x/1.4 NA objective. Staining, imaging, and quantification were performed by two blinded investigators using ImageJ software. In brief, maximum intensity projections were generated from the z-stacks and used to create a mask with the CD31 signal (green channel), then the mean intensity of Poldip2 staining (red signal) contained in the CD31 area was calculated. Results show the integrated density of Poldip2 staining (mean gray values x pixel number ± standard error of the mean [SEM]).

For mouse tissue processing, following euthanasia, lungs were instilled with 10% formalin through the trachea, excised and placed in 10% formalin for one week. Lungs were then transferred to 70% ethanol, dehydrated using a HistoCore PEARL automatic tissue processor (Leica), paraffin-embedded, and sectioned. Lung sections, 5 μm in thickness, were either stained with hematoxylin (ab220365, Abcam) and eosin (ab246824, Abcam) (H&E) for morphological analysis or processed for immunofluorescence.

H&E whole slide images were acquired with a NanoZoomer SQ instrument (Hamamatsu) with a 20x objective lens. Quantification was performed by an operator blinded to mouse genotype and treatment. Three representative tiles, measuring 500 x 500 µm, were extracted from each whole slide image, using QuPath v0.5.1 software [25] and rescaled to 680 x 680 pixels. Septal thickness was measured with a semi-automated method [26], using the morphometry_v4.0 plugin for Fiji software [27] (S1 Fig). Manual preprocessing enabled exclusion of non-parenchymal regions and minimized artifacts from large vessels or airways.

Neutrophil infiltration in mouse lungs was assessed by immunofluorescence following co-staining for the leukocyte-common antigen, CD45, and myeloperoxidase (MPO). Lung sections were covered with water and treated with UV light for 30 min in a humidity chamber to reduce autofluorescence. Antigen retrieval was performed using 10 mM citrate buffer (pH 6.0) in a pressure cooker for 10 minutes. Blocking was carried out for 1 hour using 10% goat serum and 2% IgG-free BSA in Universal Buffer (0.05 M Tris HCl, pH 7.6, 0.15 M NaCl, and 0.1% sodium azide). Consecutive sections were then incubated overnight at 4°C with primary antibodies: rabbit anti-CD45 (ab10558, Abcam) and rat anti-MPO (ab300650, Abcam), or with the corresponding IgG isotype controls (rabbit ab172730, Abcam and rat ab18450, Abcam) dissolved in blocking buffer. Next, the sections were incubated for 1 hour at room temperature with secondary antibodies: anti-rabbit Alexa Fluor 568 (A11011, Invitrogen), anti-rat Alexa Fluor 488 (A11006, Invitrogen), and DAPI (D9542, Sigma-Aldrich). To further reduce autofluorescence, samples were then incubated with 0.1% Sudan black B (TS41983−0100, VWR) in 70% ethanol for 10 minutes, washed three times with Universal Buffer, and mounted using ProLong Diamond Antifade (P36961, Thermo Fisher Scientific). At least 12 images from each slide were acquired as z-stacks using a Zeiss LSM800 Airyscan laser scanning confocal microscope with an EC Plan-Neofluar 40x/1.3 NA objective. Staining, imaging, and quantification were performed by two blinded investigators. In brief, maximum intensity projections were generated from the z-stacks and used to quantify the number of positive cells with the “Cell Counter” plugin in ImageJ software, as well as to measure the tissue area analyzed in each image. The numbers of MPO and CD45 double positive cells were normalized to total tissue area.

### RNA extraction and RT-qPCR

Mouse lung samples were homogenized in 1 mL of QIAzol reagent (79306, Qiagen) with a single stainless steel bead using a TissueLyser LT (85600, Qiagen) at 25 Hz for 10 min. In a pilot study we verified that the virus was completely inactivated by this procedure. Total RNA was purified with the RNeasy Plus kit (74134, Qiagen) or Direct-zol DNA/RNA Miniprep (R2080, Zymo Research). Reverse transcription was performed using Protoscript II reverse transcriptase (M0368, New England Biolabs) with random primers. cDNA was amplified with 2X Forget-Me-Not EvaGreen qPCR Master Mix with Low ROX (31045, Biotium) and primers against mouse TNF𝛼, IL-1β, IL-6, MCP1, CXCL1, IFN-γ, Poldip2 and human ACE2 (**Table 1**). Reactions were carried out in 96-well qPCR plates (4346907, Thermo Fisher Scientific), using a QuantStudio 7 Flex (Thermo Fisher Scientific) Real-Time qPCR System. Quantification was performed using the mak3i module of the qpcR software library [28,29] in the R environment [30]. Data were normalized using the NORMA-Gene software, which eliminates the need for housekeeping gene normalization [31]. Relative gene expression was calculated by dividing all values by the average of the Poldip2 + / + PBS control group. Thus, the average control was scaled to 1 to facilitate visual comparisons between groups.

### Statistics

Data are presented as mean ± SEM. A Log transformation was applied when required to normalize distributions, as shown by the Log scale of the Y axis in bar graphs. Human data were analyzed using a two-tailed nested t-test. Mouse viral load data were analyzed using two-tailed Mann-Whitney tests, other mouse data were analyzed using a two-tailed unpaired t*-*test for single comparisons or two-way analysis of variance (ANOVA), followed by multiple comparisons with Sidak or Fisher’s LSD test. A threshold of P < 0.05 was considered significant. Calculations were performed using Prism 10 (GraphPad).

## Results

### Endothelial Poldip2 is upregulated in human lung after SARS-CoV-2 infection

**Fig 1:**
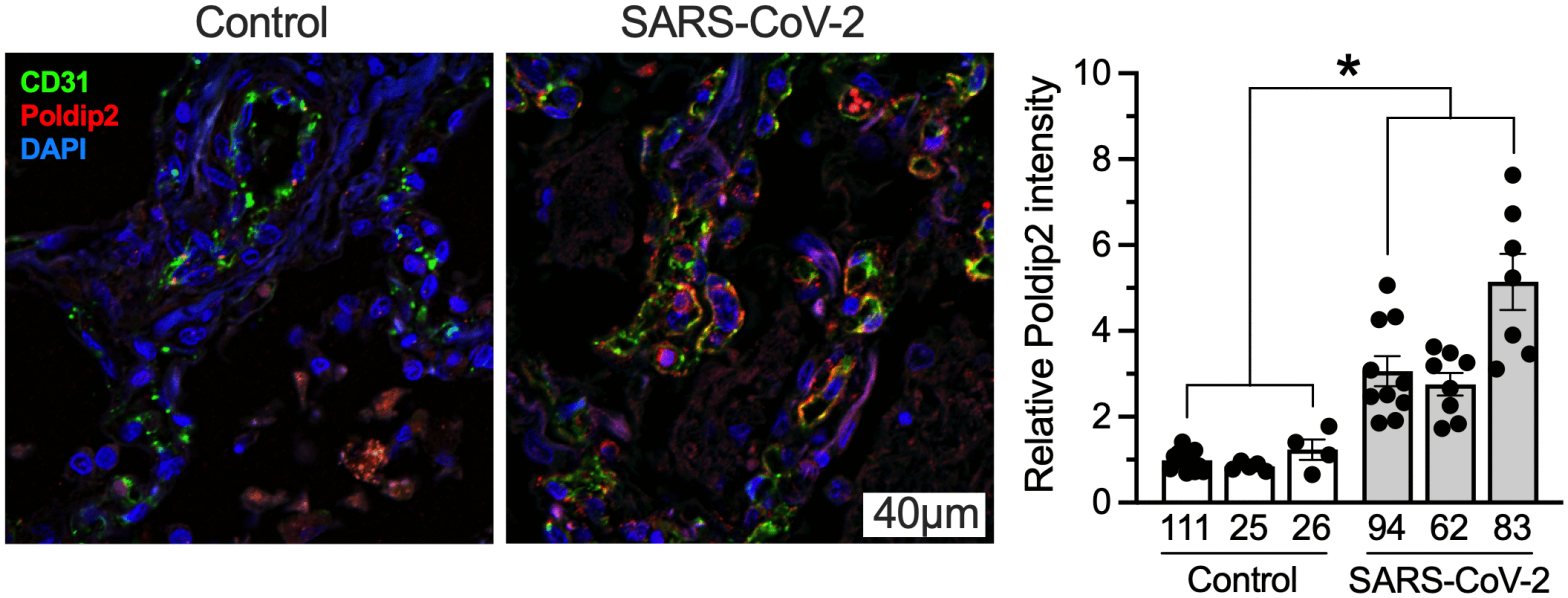
Endothelial Poldip2 is upregulated in human lung after SARS-CoV-2 infection. Sections of lung tissue were immunostained for CD31 (green), Poldip2 (red) and nuclei (blue). Left: representative images from one control and one infected patient. Right: quantification of Poldip2 staining in CD31-positive areas. Numbers below the bars correspond to individual patients. Bars represent means ± SEM from 4-10 pictures per sample taken at random locations. Data were analyzed using a two-tailed nested Student t-test, n = 3, *P < 0.05.

To begin exploring a possible contribution of Poldip2 to COVID-19 pathology, we analyzed lung tissue sections from control and virus-infected patients. Since our previous results pointed to a positive effect of Poldip2 on vascular endothelial permeability [13,16,18,19,32], we assessed Poldip2 co-expression with the endothelial marker CD31 by immunofluorescence. As shown in **Fig 1**, endothelial Poldip2 was upregulated in patients with SARS-CoV-2 infection, compared to controls. This observation suggests that one of the ways in which the virus may harm patients is via upregulation of Poldip2, which may in turn aggravate disease by increasing endothelial permeability in lung. Therefore, in the present study we investigated the potential benefit of knocking down Poldip2 in an animal model of COVID-19.

### Heterozygous deletion of Poldip2 does not affect the susceptibility to acute SARS-CoV-2 infection

**Fig 2:**
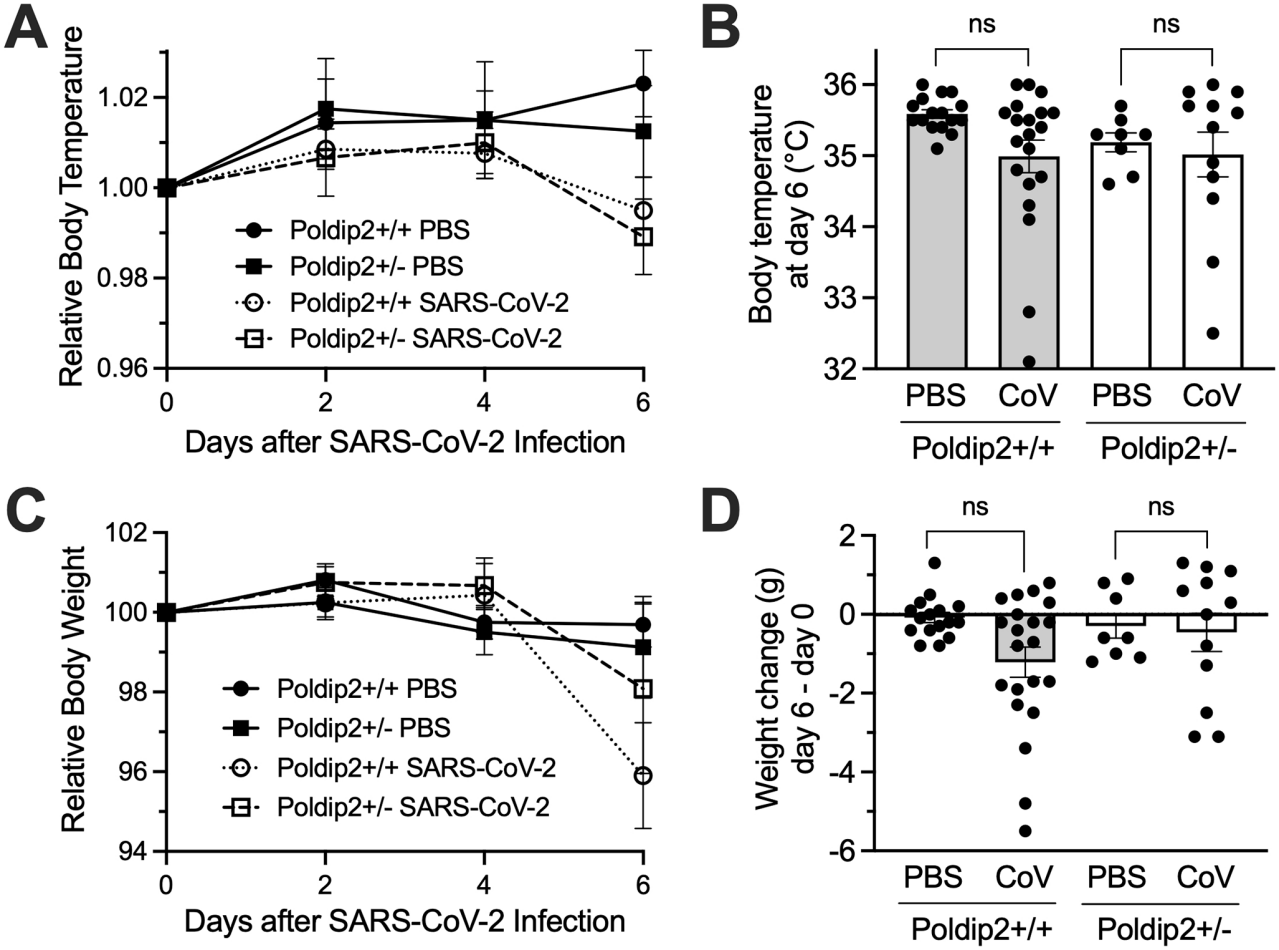
Acute infection does not significantly reduce body weights and temperatures. Male and female human ACE2 transgenic, Poldip2^+/+^ or Poldip2^+/-^ (hACE2/Poldip2) mice received a single intranasal inoculation of PBS or SARS-CoV-2 on day zero. Body temperatures (A, B) and weights (C, D) were measured every other day. Time courses (A, C) show relative values, calculated by dividing measurements for each mouse by its own initial value (day zero) and multiplied by 100 for weights. The graphs represent means ± SEM of data from n = 8-21 animals. Body temperatures at day 6 (B) and weight changes between day 6 and day zero (D), were analyzed by 2-way ANOVA: ns, not significant. On day zero, average body temperatures were identical between males and females, across all groups (35 ± 0.1°C) and average body weights were higher by a few grams in males (M) than females (F): M 29.4 ± 0.7, F 21.6 ± 0.4 in Poldip2 + /+ and M 25.9 ± 0.5, F 21.4 ± 0.3 in Poldip2 + /- (errors indicate SEM).

The next question was whether human ACE2 transgenic, Poldip2^+/+^ vs. Poldip2^+/-^ (hACE2/Poldip2) mice are equally susceptible to SARS-CoV-2 infection. Mice given a single intranasal instillation of PBS or 4x10^5^ PFU SARS-CoV-2 virus were monitored every other day for body weight and temperature changes until day 6. As indicated in **Fig 2**, SARS-CoV-2 tended to decrease body temperature in both Poldip2^+/+^ and Poldip2^+/-^ mice at day 6 post-infection, compared to PBS controls, with no difference between genotypes. These trends did not reach statistical significance, perhaps in part because the sickest animals were humanely euthanized and excluded from the study. Similarly, SARS-CoV-2 infection did not induce a significant weight loss in either Poldip2^+/+^ or Poldip2^+/-^ mice. Temperature and weight measurements in time courses were normalized to the initial value for each mouse to eliminate differences in baseline between animals. These data suggest that Poldip2^+/+^ and Poldip2^+/-^ mice are equally susceptible to SARS-CoV-2 infection.

### Heterozygous Poldip2 knockdown reduces SARS-CoV-2 burden 7 days after infection

**Fig 3:**
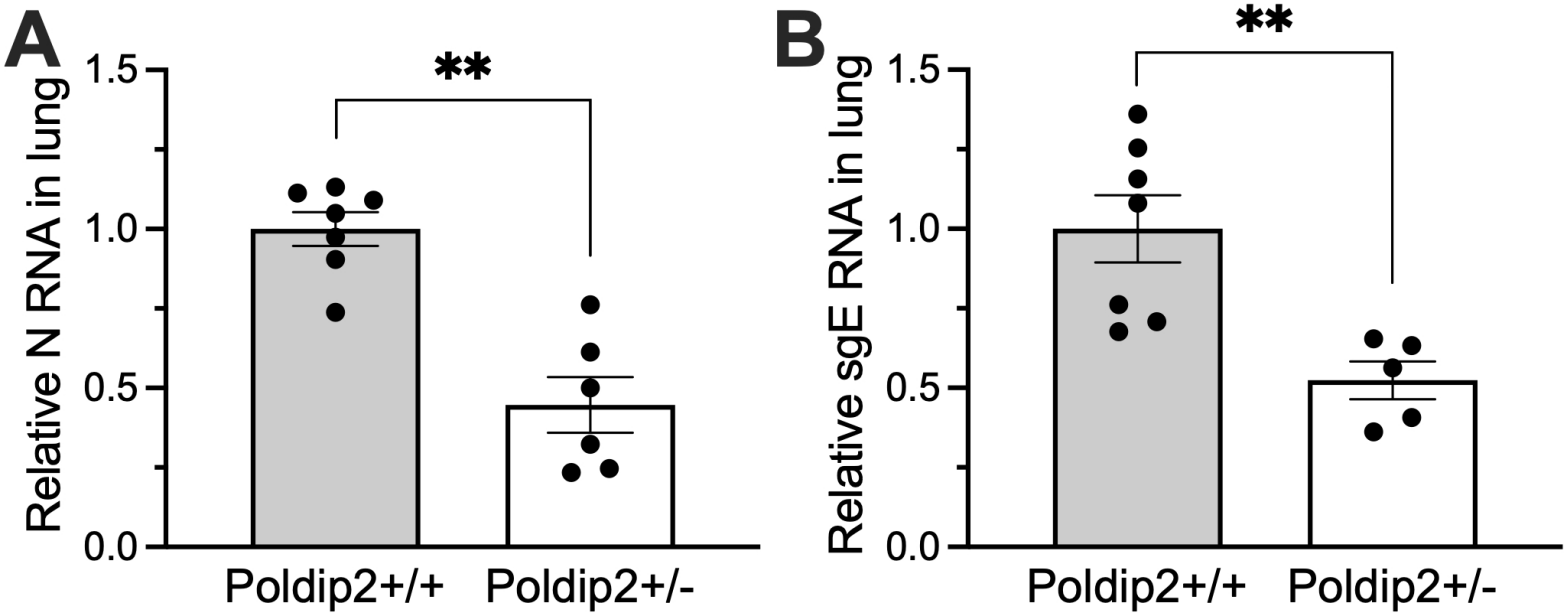
Poldip2 depletion reduces viral load 7 days post-infection. Male and female hACE2/Poldip2 mice were infected with SARS-CoV-2 by nasal inoculation. Lungs were collected 7 days later for RNA preparation. Viral loads were measured by RT-qPCR using primer pairs and TaqMan probes specific for the viral gene N (A) or subgenomic E (sgE) RNA (B). Bars represent means ± SEM of data from n = 5-7 animals. Groups were compared using two-tailed Mann-Whitney tests: **P < 0.01.

To evaluate possible longer-term consequences of the disease, hACE2/Poldip2 mice were euthanized 7 days after infection. Lungs were harvested to measure viral loads by RT-PCR. As shown in **Fig 3**, lung viral genomic and subgenomic RNAs were reduced in Poldip2^+/-^ mice compared to Poldip2^+/+^ control mice, suggesting that Poldip2 knockdown can curtail viral proliferation or accelerate recovery from the disease.

We also confirmed that Poldip2 mRNA levels were decreased by about 50% in Poldip2^+/-^, compared to Poldip2^+/+^ mice (S2A Fig) and that the expression of human ACE2 mRNA was not affected by Poldip2 depletion (S2B Fig).

### Poldip2 depletion reduces SARS-CoV-2-induced lung tissue damage

**Fig 4:**
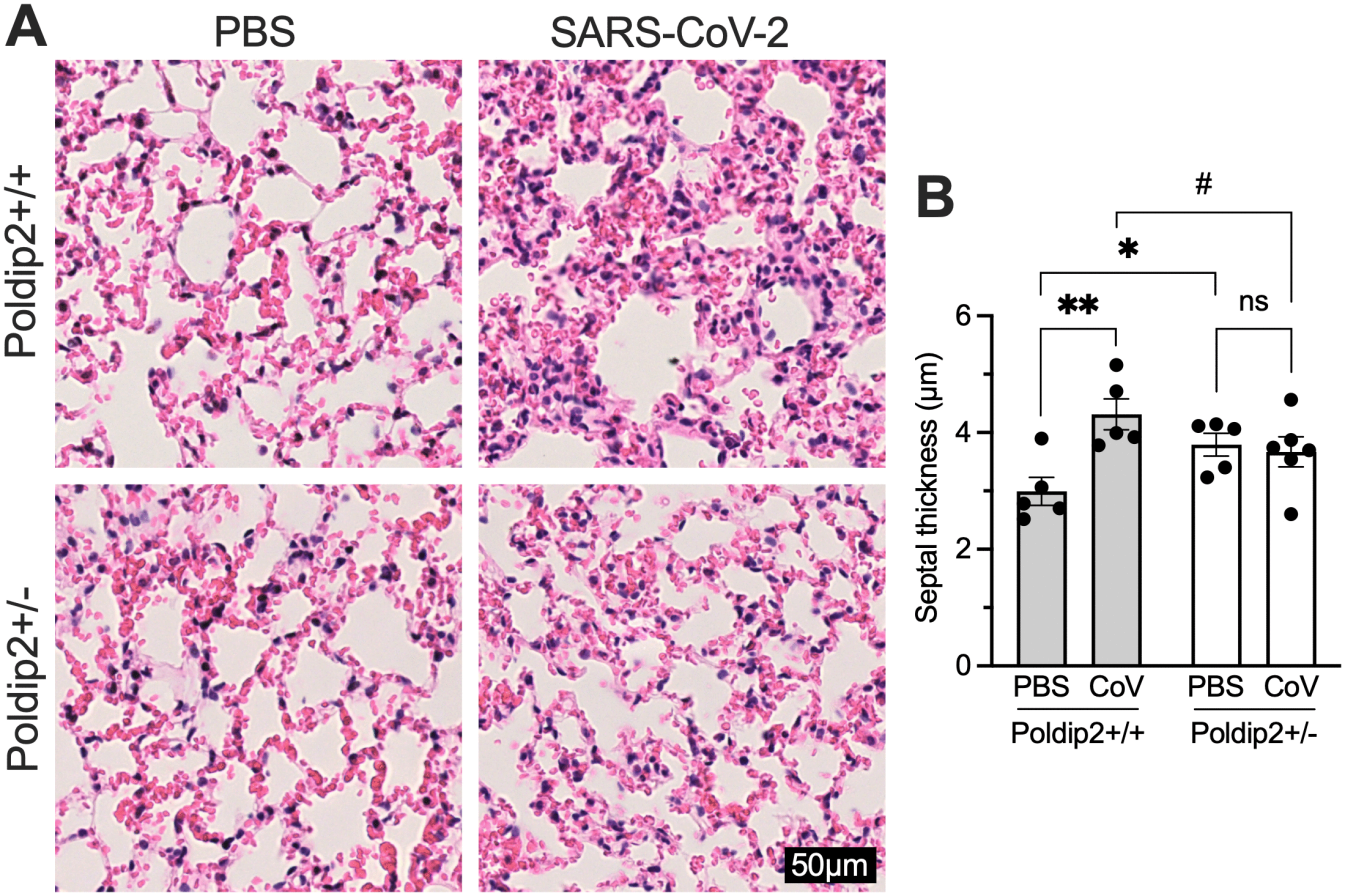
Poldip2 depletion alleviates lung tissue alterations induced by SARS-CoV-2 infection. Male and female hACE2/Poldip2 mice were euthanized 7 days after intranasal administration of PBS or SARS-CoV-2. Lungs were processed for histology and paraffin sections were stained with hematoxylin/eosin. (A) SARS-CoV-2 infection increased tissue damage and alveolar wall thickness compared to PBS in Poldip2^+/+^ (top), but not in Poldip2^+/-^ mice (bottom). (B) Septal thickness was quantified as described in Methods. Bars represent means ± SEM from n = 5-6 mice. Data were analyzed by 2-way ANOVA: ns, not significant, #P = 0.075, *P < 0.05, **P < 0.01.

Given the previously described beneficial effect of reducing Poldip2 levels in a lipopolysaccharide (LPS)-induced acute lung injury model [18,19], we sought to determine if Poldip2^+/-^ mice would exhibit similar protection after SARS-CoV-2 infection. Lungs were collected 7 days after nasal instillation of virus or saline and obvious differences in the numbers of hemorrhagic lesions were observed between infected and non-infected animals upon tissue dissection. To support these macroscopic observations, tissue sections were processed for hematoxylin and eosin (H&E) staining. Histological evaluation showed that SARS-CoV-2 caused marked lung tissue damage in Poldip2^+/+^ animals, whereas Poldip2^+/-^ mice were resistant to virus-induced lung injury (**Fig 4A**). Quantitative analysis of micrographs by an operator blinded to genotypes showed that septal thickness was increased by the virus in Poldip2^+/+^, but not in Poldip2^+/-^ animals. Furthermore, Poldip2^+/-^ mice had a higher septal thickness at baseline, which may have contributed to their resilience (**Fig 4B**).

### Poldip2 mediates neutrophil infiltration in the lungs following SARS-CoV-2 infection

**Fig 5:**
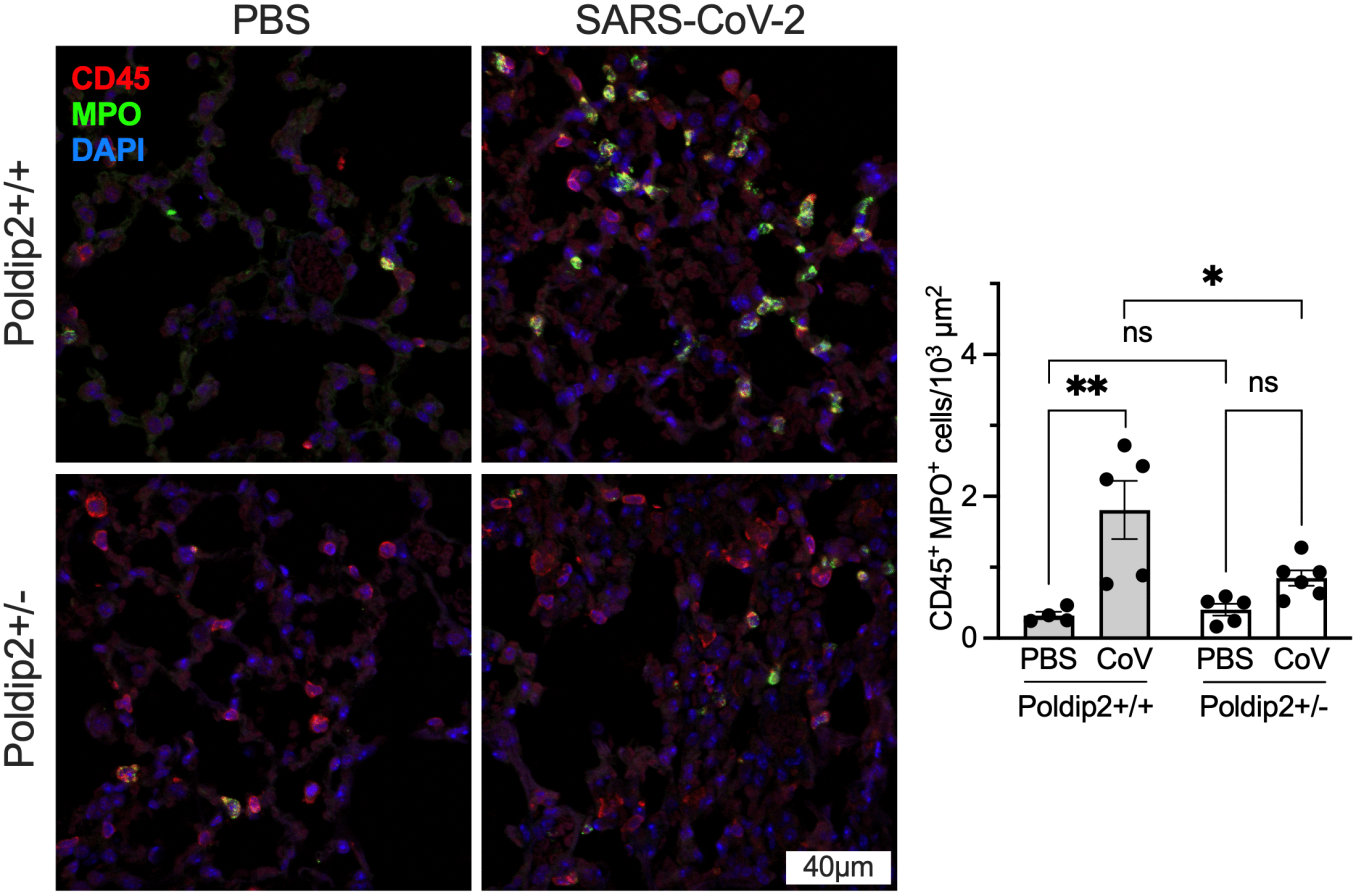
Poldip2 depletion reduces neutrophil infiltration in lung after SARS-CoV-2 infection. Lungs from male and female hACE2/Poldip2 mice were harvested 7 days after nasal PBS or virus instillation and processed for immunofluorescence. Tissue sections were stained to detect the leukocyte common antigen (CD45, red), the neutrophil marker myeloperoxidase (MPO, green) and nuclei (blue), as shown in representative micrographs (left). Cells positive for both CD45 and MPO were counted in 10-13 images per animal (right). Bars represent means ± SEM of data from n = 4-6 mice. Data were analyzed using 2-way ANOVA: ns, not significant; *P < 0.05, **P < 0.01.

Since lung injury was reduced in Poldip2^+/-^ mice, we sought to investigate if Poldip2 is involved in neutrophil recruitment into the lungs after SARS-CoV-2 infection. Lungs were collected at day 7 after PBS or virus instillation and processed for neutrophil-specific staining. Immunofluorescence data revealed a reduction in infiltrating CD45 and myeloperoxidase-positive neutrophils (**Fig 5**) in the lungs of Poldip2^+/-^ mice after SARS-CoV-2 infection, compared to Poldip2^+/+^ mice. This effect is consistent with the decrease in lung injury observed above in Poldip2 knockdown mice.

### Poldip2 depletion tends to attenuate chemokines induced by SARS-CoV-2 infection

**Fig 6:**
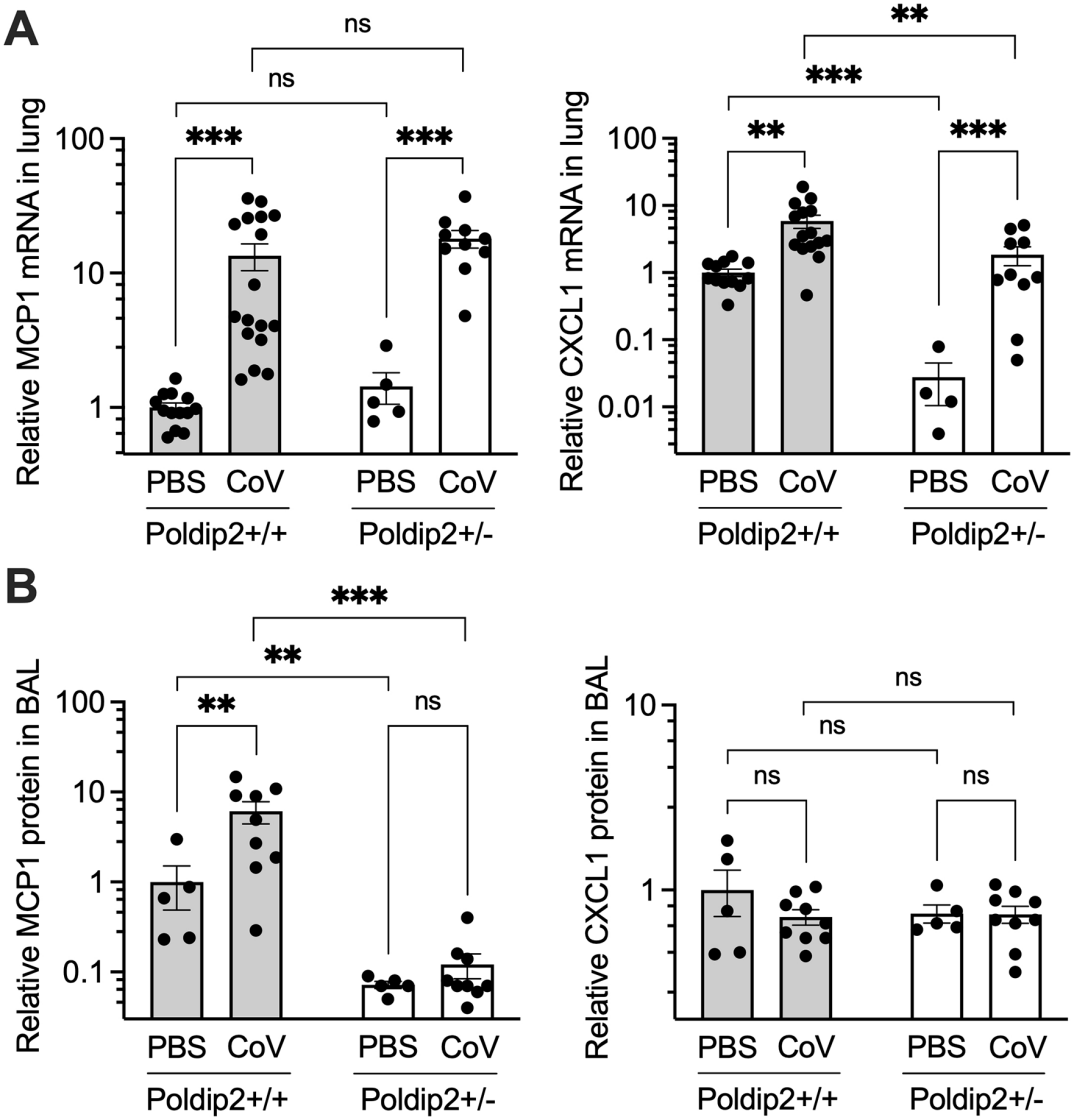
Poldip2 depletion tends to attenuate chemokine expression. Male and female hACE2/Poldip2 mice were euthanized 7 days after intranasal administration of PBS or SARS-CoV-2. Bronchoalveloar lavage (BAL) fluid was collected before harvesting lungs for RNA extraction as in Figure 3. mRNAs were measured by RT-qPCR in lung tissue (A) and protein ELISAs were carried out in BAL (B). Bars represent means ± SEM of data from n = 4-17 animals. Basal protein levels in +/ + PBS were 40 ± 20 pg/ml (MCP1) and 111 ± 31 pg/ml (CXCL1). Data were analyzed using 2-way ANOVA: ns, not significant; ** P < 0.01; *** P < 0.001.

SARS-CoV-2 infection has been shown to be associated with increased expression of several chemokines. To determine whether Poldip2 regulates chemokine induction following SARS-CoV-2 infection, we first examined CXCL1 and MCP1 (Ccl2) mRNA levels in lung tissue. Seven days after intranasal administration of SARS-CoV-2, both CXCL1 and MCP1 mRNA were significantly increased in the lungs of Poldip2^+/+^ mice. While no difference was observed in MCP1 mRNA levels between Poldip2^+/+^ and Poldip2^+/-^ mice, CXCL1 baseline mRNA levels were markedly reduced in Poldip2^+/-^ animals (**Fig 6A**). In contrast, Poldip2 depletion completely prevented increases in MCP1 protein levels in bronchoalveolar lavage (BAL) fluid, while CXCL1 protein levels appeared to be similar across groups (**Fig 6B**). However, it is possible that an increase in CXCL1 by the infection might have been missed due to partial degradation of the protein during the incubation of BAL samples at 56°C for virus inactivation. In spite of those apparent differences in expression between tissue mRNA and BAL protein, these data support the idea that upregulation of chemokines by Poldip2 may contribute to leukocyte recruitment after the viral infection.

### Poldip2 depletion affects cytokine expression in lung and BAL fluid after SARS-CoV-2 infection

**Fig 7:**
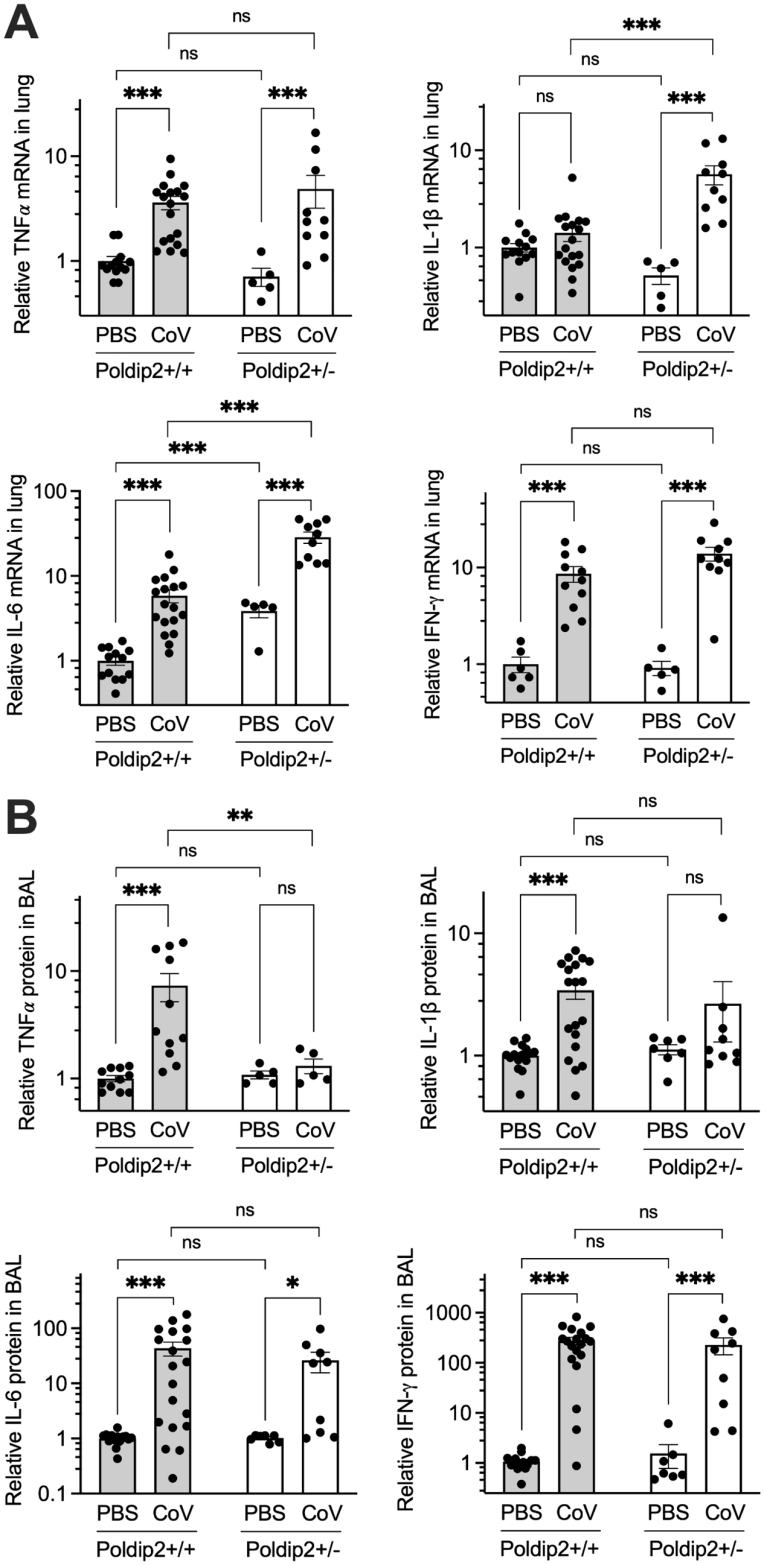
Poldip2 depletion variably affects cytokine expression after SARS-CoV-2 infection. Male and female hACE2/Poldip2 mice received a nasal instillation of PBS or SARS-CoV-2. BAL fluid and lungs were collected after 7 days and processed as in Figure 6 to measure the expression of cytokine mRNAs in lung tissue (A) and proteins in BAL (B). Bars represent means ± SEM of data from n = 5-19 animals. Basal protein levels in +/ + PBS were 1.7 ± 0.1 pg/ml (TNFα), 5.0 ± 0.3 pg/ml (IL-1β), 11.8 ± 0.8 pg/ml (IL-6), 4.2 ± 0.5 pg/ml (IFN-γ). Data were analyzed using 2-way ANOVA: ns, not significant; * P < 0.05; ** P < 0.01; *** P < 0.001.

To determine whether Poldip2 also regulates cytokine induction after SARS-CoV-2 infection, we examined mRNA expression in lung tissue and protein in BAL fluid, 7 days after nasal instillation of PBS or SARS-CoV-2 in Poldip2^+/+^ and Poldip2^+/-^ mice. Although TNFα and IFN-γ mRNAs were significantly upregulated in Poldip2^+/+^ mice, they were not affected by Poldip2 depletion. Interestingly, upregulation of both IL-1β and IL-6 mRNAs were unexpectedly exacerbated in Poldip2^+/-^, compared to Poldip2^+/+^ mice in response to SARS-CoV-2 (**Fig 7A**). Additionally, IFN-γ and IL-6 protein levels in BAL were increased in both Poldip2^+/+^ and Poldip2^+/-^ mice and no difference was found between the two genotypes. In contrast, TNFα and IL-1β protein levels were increased after SARS-CoV-2 in BAL of Poldip2^+/+^ mice, but not in Poldip2^+/-^ animals (**Fig 7B**). These observations suggest that Poldip2 may modulate the inflammatory response to SARS-CoV-2 in a heterogeneous manner, reflecting its diverse regulatory roles.

## Discussion

The present study revealed that Poldip2 is upregulated in the lungs of COVID-19 patients and assessed the potential benefit of targeting Poldip2 in a mouse model. At day 7 following viral infection in mice, Poldip2 knockdown alleviated viral burden, lung tissue damage and neutrophil infiltration. The beneficial effects of Poldip2 depletion were most notable in BAL fluid, with blunted induction of MCP1 and TNFα. Therefore, our data support the idea that Poldip2 contributes to the pathology induced by SARS-CoV-2 and warrant further study of its functions.

In humans, SARS-CoV-2 infection presents a broad clinical spectrum ranging from asymptomatic to severe illness [33]. Several animal models that recapitulate the clinical and pathological characteristics of COVID-19 have become invaluable tools for elucidating the biological pathways underlying SARS-CoV-2 infection [2]. Because the spike protein of the initial SARS-CoV-2 strain fails to engage murine ACE2, in this study we used a transgenic mouse model in which the human ACE2 is overexpressed under the K18 epithelial promoter. Thus, human ACE2 is expressed in airway epithelial cells, colon and to a lesser extent kidney, liver, spleen, and small intestine [34,35]. Therefore, these transgenic mice are susceptible to SARS-CoV-2 infection and develop acute respiratory disease after intranasal exposure, reproducing the major elements of severe disease observed in humans [35]. Previous studies using K18-hACE2 mice have demonstrated that intranasal exposure to 2 different doses of SARS-CoV-2, 2 × 10^3^ or 2 × 10^4^ PFU, resulted in acute disease with considerable weight loss (12%−20%) and lung injury, with some animals at the lower dose surviving infection despite significant weight loss [35]. A different study using the same animal model has shown that mice infected with 2.5 × 10^4^ PFU presented a 25% weight loss by day 7 after infection, which was less pronounced and delayed in mice infected with a lower dose, 1 × 10^2^ PFU [36], suggesting dose-dependent SARS-CoV-2 manifestations. In our study (using 4x10^5^ PFU), starting on day 4 Poldip2^+/+^ K18-hACE2 animals began to show signs of SARS-CoV-2 infection, presenting trends towards decreases in body temperature and weight loss, but to a lesser extent than in published studies. This difference may be due to a reduced viral titer after storage or to greater supportive care of our experimental animals. However, the most severely affected animals were excluded from our study because they died before day 7 or were humanely euthanized. In our hands, all infected mice, regardless of genotype, exhibited a similar body temperature and weight loss tendency in response to SARS-CoV-2.

Our previous studies indicated an important role of Poldip2 in modulating cytokine and chemokine induction in an LPS-induced acute lung injury model [18,19]. Heterozygous deletion of Poldip2 was protective against ARDS-induced TNFα, MCP1, IL-1β and CXCL1 in lung tissue. In addition, we recently found that endothelial-specific Poldip2 deletion *in vivo* also results in reduced expression of proinflammatory cytokines and chemokines, including TNFα, CXCL1, CXCL2, IL-1β and IL-6, following LPS-induced ARDS [18]. The current study points to a new mechanism implicating Poldip2 in disease. Infection by SARS-CoV-2 appears to upregulate Poldip2 in lung vascular endothelium (Fig 1), which may exacerbate vessel permeability, tissue invasion by the pathogen and aggravation of the disease. Future work will be required to assess whether Poldip2 is also upregulated in other tissues and by different pathogens. Conversely, reduced endothelial permeability caused by Poldip2 deficiency may not only help control edema, but also impair viral dissemination into tissues, curtail viral proliferation and accelerate recovery. This idea is consistent with the lower viral burden observed in Poldip2 + /- mice 7 days after infection (Fig 3). However, determining whether this effect resulted from decreased tissue invasion, accelerated viral clearance, or other mechanisms will require further study.

Because Poldip2 seemed to have a profound effect in promoting the inflammatory response in our previous work, we hypothesized that Poldip2 depletion would also be protective after SARS-CoV-2 infection. In this study, SARS-CoV-2 led to a pronounced inflammatory response in lung tissue with upregulation of several pro-inflammatory cytokines and chemokines, including TNFα, IL-6, IL-1β, IFN-γ, MCP1, and CXCL1. Consistent with our previous studies using the LPS-induced ARDS model [19], we found that heterozygous deletion of Poldip2 significantly reduced TNFα, MCP1, and CXCL1 induction in BAL or lung tissue following SARS-CoV-2. Interestingly, both IL-6 and IL-1β mRNA levels were significantly upregulated in lung tissue of Poldip2^+/-^, compared to Poldip2^+/+^ mice, suggesting that loss of Poldip2 has a complex modulatory effect on the inflammatory response triggered by viral infection.

Given the previously described effect of Poldip2 on leukocyte attachment and infiltration into inflamed tissues [17–19], we sought to investigate the contribution of Poldip2 in leukocyte recruitment into the lungs after SARS-CoV-2 infection. Several studies have reported massive immune cell infiltration in the lungs during severe COVID-19 infection, with enhanced recruitment of myeloid cells [36,37] and changes in lymphoid cells, such as T cells [38] and natural killer cells [39]. However, we still lack comprehensive insights into the immunopathology of post–severe COVID-19 infection in lung tissue. Our data show that SARS-CoV-2 led to pronounced MPO-positive neutrophil infiltration into the lungs, which was largely prevented by heterozygous deletion of Poldip2. This could be explained by the reduced basal levels of the chemoattractant CXCL1 which has been shown to induce neutrophil recruitment [40]. However, it is also possible that neutrophils from Poldip2^+/-^ mice are not fully attaching to the inflamed endothelium, as our previous study has shown that Poldip2 mediates β2‐integrin activation during neutrophil recruitment to inflamed lungs [41]. Corroborating our data, other studies have also demonstrated that infected K18-hACE2 mice presented a pronounced increase in MPO-positive neutrophils in lung tissue upon exposure to SARS-CoV-2 [35]. Neutrophil extracellular traps, which are indicative of neutrophil activation and can contribute to inflammation-associated lung damage, have also been described in lung autopsy samples [42], highlighting the importance of neutrophil recruitment as a potential therapeutic target in severe COVID-19. Of note, the germicidal function of neutrophils is likely not affected in Poldip2 + /- mice because they appeared to be fully functional in our previous *in vitro* assays that evaluated extracellular trap formation, reactive oxygen species and cytokine production [41].

It is also worth noting that in the current study, mice were only infected with one particular strain of SARS-CoV-2. This strain was originally isolated from a patient in the U.S. state of Washington in 2020 (see Methods), before numerous variants started to emerge around the world. It is possible that the effect of Poldip2 knockdown varies between viral strains with distinct pathogenic and inflammatory profiles. However, that question was not addressed here and will require further study.

Exacerbated immune responses play a major role in the pathophysiology of SARS-CoV-2, leading to severe lung injury [43]. Together, our results show that Poldip2 depletion decreases viral burden, neutrophil infiltration and lung tissue damage following SARS-CoV-2 infection. Thus, our study reveals a potential beneficial effect of Poldip2 depletion in SARS-CoV-2 infection and further adds to our understanding of the detrimental role of Poldip2 on endothelial barrier function under inflammatory conditions. In addition, the identification of molecular mechanisms driving severe pathogenic processes in the lung may provide critical insight into the molecular and cellular features of COVID-19 pathogenesis.

## Supporting information

S1 Fig Image segmentation to measure alveolar septal thickness. Lungs from male and female hACE2/Poldip2 mice were processed and stained with H&E. Example of a 500 x 500 µm tile extracted from a whole slide image (Left). Corresponding binary mask generated by the morphometry Fiji plugin, showing successful segmentation prior to measurement of septal thickness (Right). Additional details can be found under Methods and in the original publication [26].
(TIF)

S2 Fig Expression of mouse Poldip2 and human ACE2 mRNAs. Lungs were collected from male and female hACE2/Poldip2 mice and processed to measure mRNAs by RT-qPCR as in Figure 3. As expected, Poldip2 expression was reduced by about 50% in Poldip2^+/-^, compared to Poldip2^+/+^ controls (A). In addition, the expression of human ACE2 mRNA was not affected by the Poldip2 genotype (B). Bars represent means ± SEM of data from 9 animals. Means were compared using an unpaired two-tailed Student t-test: ns, not significant; *** P < 0.001.
(TIF)
